# Supplementary material for: Fat: Quality, or Quantity? What Matters Most for the Progression of Metabolic Associated Fatty Liver Disease (MAFLD)
Source: Biomedicines. 2021 Sep 22;9(10):1289. doi: 10.3390/biomedicines9101289 (PMC8533605; doi:10.3390/biomedicines9101289)
Supplement: Supplementary file 1 [file biomedicines-09-01289-s001.zip › biomedicines-1359641-supplementary.pdf]

Supplementary Material:

Fat quality or quantity? What matters most for the progression of Metabolic Associated Fatty Liver Disease (MAFLD)

Olga Estévez-Vázquez, Raquel Benedé-Ubieto, Feifei Guo, Beatriz Gómez, Patricia Aspichueta, Johanna Reissing, Tony Bruns, Carlos Sanz-García, Svenja Sydor, Lars P. Bechmann, Eva Maranillo, José Ramón Sañudo, María Teresa Vázquez, Arantza Lamas-Paz, Laura Morán, Marina S. Mazariegos, Andreea Ciudin, Juan M Pericàs, María Isabel Peligros, Javier Vaquero, Eduardo Martínez-Naves, Christian Liedtke, José R. Regueiro, Christian Trautwein, Rafael Bañares, Francisco Javier Cubero, Yulia A. Nevzorova

TABLE OF CONTENT

Fat quality or quantity? What matters most for the progression of Metabolic Associated Fatty Liver Disease (MAFLD) 1
SUPPLEMENTARY METHODS 2
Animal Experimentation 2
Glucose Tolerance Test (GTT) 2
Histological analysis 3
Immunofluorescence (IF) staining 3
Immunohistochemistry (IHC) staining 5
Image analysis 6
RNA isolation and RT-qPCR 6
Western blot 7
Lipid extraction and quantification 7
Magnetic resonance imaging (MRI) 7
SUPPORTING TABLES 9
Supplementary figure legends 13
Supplementary References 13

SUPPLEMENTARY METHODS

Animal Experimentation

10 weeks C57BL/6J male mice were randomly distributed into 4 groups with sample size of 7 to 9 animals per group. A total sample size of 29 was based on a priori power analysis (a statistical power of 0.95, a specific α error probability of 0.05 and effect size= 0.744) in our primary outcome measures (liver weight) in treated mice. Analysis was performed by Using G*Power analysis software version 3.1.9.4, Franz Faul, Universität Kiel, Germany (https://www.psychologie.hhu.de/arbeitsgruppen/allgemeine-psychologie-und-arbeitspsychologie/gpower) [1].

Body weight was recorded every 7 days. Upon sacrifice mice were fasted for 12 h and sacrificed using isoflurane (Solvet, Spain). Blood from the vena cava was collected and serum parameters of Alanine Aminotransferase (ALT), Aspartate Aminotransferase (AST), Lactate Dehydrogenase (LDH) and cholesterol were analyzed by automated analyzers in the University Hospital RWTH Aachen, (Aachen, Germany). Liver histology and molecular analysis were performed as described below. Liver histology was evaluated by an expert liver pathologist (M.I.P.) blinded to the dietary condition.

Glucose Tolerance Test (GTT)

At the end of the treatment, GTT was performed after 12 h fasting through an Intraperitoneal (IP) injection of 7.5 g/kg body mass of 20% glucose solution (Braun GmbH, Krönberg, Germany). Glucose levels were measured using an Accu-Check glucometer (Roche, Germany) at 0, 5, 30, 60, 90 and 120 minutes after the injection as previously described [2].

Histological analysis

Liver and eWAT histology were assessed by Hematoxylin and Eosin (H&E) and Sirius Red (SR) stainings in 5 µm paraffin embedded sections using the standard protocols. The presence of steatosis was further confirmed by Oil Red O (ORO) staining in 7 µm frozen sections and quantified as previously described [3].

Immunofluorescence (IF) staining

The source of the commercially available antibodies used for the IF is listed in **S-4.**

a. F4/80, CD45

5 µm liver sections were cut from cryoblock embedded tissue in Tissue- Tek® (Sakura, Japan). Sections were defrosted for 30 min at room temperature (RT) and fixed with 4% formaldehyde (PFA) for 15 min, followed by 3x5 min washing by PBS. Afterwards, the sections were blocked with 5% goat serum (Promocell, Barcelona), diluted in PBS for 1h 30 min at RT. After removing the blocking solution, the sections were incubated with primary antibody for 1h at RT. Later, the sections were washed 3x5 min with PBS, followed by the incubation with corresponding secondary antibody (in 3% goat serum) at RT for 1 hour. Then sections were washed 3x10 min with PBS and were counterstained with mounting medium with DAPI (Vector LABS, Burlingame, USA).

b. Ki67

5 µm liver sections, were cut from cryoblock embedded in Tissue- Tek®, and defrost during 30 min at RT. Then they were fixated for 15 min in 4% PFA followed by 3x5 min washed with PBS. Sections were blocked for 45 min with blocking solution 5% BSA (Sigma- Aldrich, Germany), 0.3% Triton X-100 in 10 ml PBS.

Blocking solution was removed and sections were incubated with primary antibody (1:100) in blocking solution + 5% goat serum at 4% overnight. The following day the sections were washed with PBS 3x10 min and then incubated at RT for 1 hour with secondary anti-rabbit antibody (1:500 in 5% goat serum). After incubation, the sections were washed 3x10 min with PBS and counterstained with mounting medium with DAPI (Palex Medical, Madrid).

c. Collagen I

5 µm liver sections, were cut from cryoblock embedded in Tissue- Tek® (Sakura, Japan), and defrost during 30 min at RT. Then sections were fixed in PFA 4% for 15 min, followed by 3x5 min washed with PBS. Sections were incubated with blocking solution (5% BSA, 0.3% Triton X-100 in PBS) 1 hour RT. After they were incubated overnight at 4ºC in a wet box, with primary antibody (dilution 1:200 in blocking solution- 1% BSA, 0.3% Triton X-100). The following day, sections were washed with PBS 5x10 min. Then they were incubated with secondary antibody 1h at RT, donkey anti-rabbit 1:400 in 1% BSA, 0.3% Triton X-100. Later they were washed in PBS 3x10 min and were mounted with DAPI (Palex Medical, Madrid).

d. Phalloidin

5 µm paraffin embedded sections were dried at 65ºC for 30 min and then fixed with xylene (2x10 min). Then slides were dehydrated by decreasing percentages of ethanol: 100% 2x5 min, 95% 2x5 min, 70% 2x5 min and destinated H_2O 2x5 min, shaking. Next slides were washed 5 min in PBS, later blocking solution was added (5% goat serum in PBS). After removing the blocking, the incubation with Phalloidin was performed (in 0.1% BSA in PBS) for 30 min. Later slides were washed 3x5 PBS and briefly washed with distilled H_2O. Finally, slides were mounted with DAPI mounting media (Palex Medical, Madrid).

e. TUNEL staining

5 µm liver sections, were cut from cryoblock embedded in Tissue- Tek® (Sakura, Japan) and fixated for 20 min in 4% PFA followed by washing in PBS. After that slides were incubated in 30% H_2O_2 and Methanol (1:9) for 10 min, followed by 10 min wash with PBS. To have access to the DNA in the nuclei slides were permeabilized in 150 mM Na-citrated, 10% Triton 10x in PBS for 2 min. The solution was removed and slides were washed by 2x5 min in PBS. Next, the sections were incubated overnight at 4ºC with TUNEL labelling solution (Roche, Germany). prepared according to the standard manufacturer protocol. The next day, the sections were washed 4x10 min with PBS and mounted with DAPI media (Palex Medical, Spain). for nuclear counterstaining.

Immunohistochemistry (IHC) staining

5 µm paraffin sections were dried at 65ºC for 30 min and then fixed with xylene (2x10 min). Then slides were dehydrated by decreasing percentages of ethanol. Antigen unmasking was performed by using retriever pressure cooker with automatic program settings, by using 1mM sodium citrate acid buffer (pH=6). After cooling down the sections, they were rinsed up by distilled H2O and incubated for 10 min by using BLOXALL blocking solution (VECTOR, California). Sections were washed 2x5 min in distilled H2O, followed by a 5 min wash with PBS. Following blocking with horse serum (VECTOR, California) for 30 min at RT, sections were incubated over night with primary antibody (dilution 1:500, S-5) at 4ºC in a humid atmosphere. The following day, slides were washed with PBS, 3x5 min and the corresponding secondary antibody was applied for 1 hour at RT, in humidifying box. Next, the slides were washed 3x5 min with PBS and incubated at RT for 2-4 min with ImmPACT DAB solution * (VECTOR, California),

the reaction was stopped by introducing the sections in distilled H2O. The sections were counterstained to visualize the cellular nuclear by using Haematoxylin solution (PanReac, Spain) for 30 seconds, followed by washed with tap water up to 5 min. To dehydrate the sections, slides were introduced in ascending ethanol concentration tanks. Finally, the slides were mounted with a coverslip using Roti®- Histokitt (Carl Roth, Germany). The source of the commercially available antibodies used for the immunohistochemistry is listed in S- 5.

Image analysis

Photomicrographs of stained sections were randomly taken at 10x, 20x and 40x total magnification with an optical or fluorescence microscope as appropriate (Bright Field Microscope DM1000, Leica, Wetzlar and ZEISS Axio Lab A1. Carl Zeiss Microscopy GmbH, Jena). Seven photos from each mouse were quantified. Positive areas were determined using Image J software (http://imagej.nih.goc/ij/, National Institutes of Health, Bethesda, MD).

RNA isolation and RT-qPCR

RNA was isolated from cryopreserved liver tissue using TRIzol (Thermo Fisher, Madrid, Spain). Pure RNA was retro-transcribed to cDNA through High Capacity cDNA Reverse Transcription Kit (Thermo Fisher, Madrid, Spain) and mRNA relative expression to Glyceraldehyde-3-Phosphate-Dehydrogenase (GAPDH) was analyzed by qPCR by using a Real Time PCR System 7300 (Applied Biosystems) and Fast SYBR Green Master Mix qPCR (Thermo Fisher). Relative expression was calculated using the $2^{-\Delta\Delta Ct}$ quantification formula normalizing each gene with the expression of GAPDH, using as an internal standard [4].

Western blot

Protein content was measured using Protein Assay Dye Reagent Concentrate (Bio-Rad, California, USA) according to manufacturer's protocol. Samples were separated in 6% or 10% SDS- polyacrylamide gel and blotted into a immobilon transfer membrane (Sigma, Merck) by transference blotting. Membranes were then blocked and incubated with primary and then secondary antibodies **(S-7).** Enhanced Chemiluminescence (ECL Prime Western Blotting Detection Reagents; Amersham, Cytiva) method was used to detect protein. Quantification of Western blot was performed by densitometry using Image J software.

Lipid extraction and quantification

Lipid content was measured from 1 mg of protein (Folch J et al, 1957). Then, a separation by Thin Layer Chromatography (TLC) was done and quantified as described (Ruiz & Ochoa, 1997). For the quantification, the TLC silica-gel plates were stained with a solution of 10% $CuSO_4$ (w/v) in 8% H_3PO_4 (v/v) and an image of the plate was digitalized with GS-800 densitometer (Bio-Rad Laboratories, USA). Quantity One software (Bio-Rad Laboratories, USA), was use for quantification. A part of the lipid extract was dissolved in isopropanol (Scharlau Chemicals, Spain) and Triglycerides (TGGs) were measured using a commercial kit (Spinreact) following manufacturer´s protocol.

Magnetic resonance imaging (MRI)

The whole study has been carried out on an MRI Biospin 7T equipment (Bruker, Germany) using a volume antenna. Two sequences (T1_RARE- Rapid Acquisition with Refocused Echoes), are acquired with and without fat suppression. The parameters of these sequences are: TE / TR = 6.5 / 1500 ms.

2 averages. Rare factor 4. Coronal. Select the number of slices and adjust to the size of the animal. Fov = 80x40 mm. Matrix 256x256 pixels. Slice thickness = 0.5 mm. Slice gap = 0.25 mm. The estimated value of fat in the body, was obtained by using ImageJ (http://imagej.nih.goc/ij/, National Institutes of Health, Bethesda, MD) software. After a noise filtering process, the volume of segmented fat is measured and, using a density of 0.9 g / ml, and an estimation of the fat mass is obtained.

After homogenizing the magnetic field, a proton spectrum of the entire body of the animal is acquired using a PRESS_1H sequence with the following parameters: TE / TR = 16.5 / 2500 ms and 64 averages.

The analysis was carried out with the MNova software (Mestrelab, Spain). The area under the curve (AUC) of the water and fat peaks was measure, as well as the intensity of these peaks, and the relationship between peaks of these values is calculated.

SUPPORTING TABLES

Supporting Table S1 (S-1) Control and WDs compositions

		CTRL LASQdiet® Rod18	LP-WD D16010101	HP-WD D18121807	HP-Trans-WD D09100301
Main nutrients (kcal%)	Proteins	18,9	20	20	20
	Fat	5,3	40	40	40
	Carbohydrates	76	40	40	40

Supporting Table S2 (S-2) Main Ingredients of WDs

% Diet	LP-WD D16010101	HP-WD D18121807	HP-Trans-WD D09100301
**Corn Oil**	15	10	0
**Soybean Oil**	2.8	2.8	2.8
**Palm Oil**	0	5	0
**Primex Shortening (Trans-fat)**	0	0	15.9
**Lard**	2.2	2.2	2.2
**Fructose**	22.1	22.1	22.1
**Cholesterol**	2	2	2

Supporting Table S3 (S-3) Concentration of PA in WDs

% Diet	LP-WD	HP-WD	HP-Trans-WD
**Total Fat (g)**	180	180	180
**C16, Palmitic acid (PA)(g)**	13.6	30.3	29.8

Supporting Table S4 (S-4) Antibodies used for IF staining

ANTIBODY	REFERENCE/COMPANY	DILUTION
Rat Anti-mouse CD45	550539 BD; Pharmingen (San Diego, CA)	1:150
Rat Anti-mouse F4/80	MGCA497GA; BIO-RAD (Hercules, CA)	1:150
Ki67	Ab16667; Abcam, SP6 (Burlingame, CA)	1:100
Phalloidin	P5282; Sigma-Aldrich (St. Louis, MO)	1:500
Primary antibody Anti-mouse Collagen I	Ab34710; Abcam (Burlingame, CA)	1:200
Alexa Fluor 488 goat anti-rat IgG (H+L)	A11006; Invitrogen (Paisley, UK)	1:300
Alexa Fluor 488 donkey anti-rabbit IgG (H+L)	A21206; Invitrogen (Paisley, UK)	1:400

Supporting Table S5 (S-5) Antibodies used for IHC staining

ANTIBODY	REFERENCE/COMPANY	DILUTION
Rabbit Anti Mouse α-SMA	Ab32575; Abcam (Burlingame, CA)	1:100
HRP Anti-Mouse IgG (Peroxidase) Polymer Detection Kit made in horse	MP-7402; Vector LABS (Maravai LifeSciences)	-

Supporting Table S6 (S-6) Primer sequences for RT-qPCR.

Target gene	FORWARD (5' – 3')	REVERSE (5' – 3')
Gapdh	CAAGGTCATCCATGACAACT TTG	GTCCACCACCCTGTTGCTGTA G
Acc	CTGAGATTGAGGTAATGAAG ATGG	AGCCTGTTGAACTTTACTGGG
Acox	TAACTTCCTCACTCGAAGCC A	AGTTCCATGACCCATCTCTGT C
Apob	TCCAGGTACGAACTCAAGC	CACGGTATCCAGGAACAACTC
Pparγ	CACAATGCCATCAGGTTTGG	GCTGGTCGATATCACTGGAGA TC
Scd1	GTTCCAGAATGACGTGTACG A	GGCTTGTAGTACCTCCTCTG
Tnf- α	CCTCTTCTCATTCCTGCTTGT GG	GAGAAGATGATCTGAGTGTGA GG
Srebp1	ACAGTGACTTCCCTGGCCTA T	GCATGGACGGGTACATCTTCA A

Supporting Table S7 (S-7). List of antibodies used for WB.

ANTIBODY	REFERENCE/COMPANY	DILUTION
GAPDH	MCA4739, 6C5; BIO-RAD (Hercules, CA)	1:5000

CPT1-c	Sc-514555; Santa Cruz Biotechnology (Dallas, TX)	1:500
FASN	3189S; Cell Signaling	1:1000
HSC-70	Sc-7298; Santa Cruz	1:250
Anti-Rabbit IgG	7074S; Cell Signaling, HRP-linked antibody	1:3000
Anti- Mouse IgG	STAR 207p, IgG (HL):HRP; BIO-RAD	1:5000

Supplementary figure legends

Suppl. Fig. S1. Visceral obesity in mice fed with WDs. (A) Representative Magnetic Resonance Imaging (MRI) of mice after 14 weeks of feeding with different types of WD and control diet. The quantitative data represent fat/body ratio (%) means (n= 2-3). **(B)** Size measurement of adipocytes in eWAT tissue (arbitrary units) stained in H&E. **(C)** Area under the curve GTT (arbitrary units) after 14 weeks. (n= 3-5).

Suppl. Fig. S2. (A) Pathological evaluation of hepatic steatosis in mice fed with WDs (n= 4).

Suppl. Fig. S3. (A) *Tnf-α* mRNA relative expression to *Gapdh* after 14 weeks on diet (n= 4-6). **(B)** Pathological evaluation of hepatic fibrosis (n=4).

Suppl. Fig. S4 Reduction of body (A) and liver weight (g) (B) after WD withdrawal (n= 5).

Suppl. Fig. S5 Schematic representation of the hepatic and metabolic phenotypes in mice treated with different types of WD (created with BioRender.com).

Supplementary References

1. Benede-Ubieto R, Estevez-Vazquez O, Ramadori P, Cubero FJ, Nevzorova YA. Guidelines and Considerations for Metabolic Tolerance Tests in Mice. *Diabetes Metab Syndr Obes.* 2020;13:439-450.
2. Mehlem A, Hagberg CE, Muhl L, Eriksson U, Falkevall A. Imaging of neutral lipids by oil red O for analyzing the metabolic status in health and disease. *Nat Protoc.* 2013;8(6):1149-1154.
3. Nevzorova YA, Hu W, Cubero FJ, et al. Overexpression of c-myc in hepatocytes promotes activation of hepatic stellate cells and facilitates the onset of liver fibrosis. *Biochim Biophys Acta.* 2013;1832(10):1765-1775.
4. Livak KJ, Schmittgen TD. Analysis of relative gene expression data using real-time quantitative PCR and the 2(-Delta Delta C(T)) Method. *Methods.* 2001;25(4):402-408.

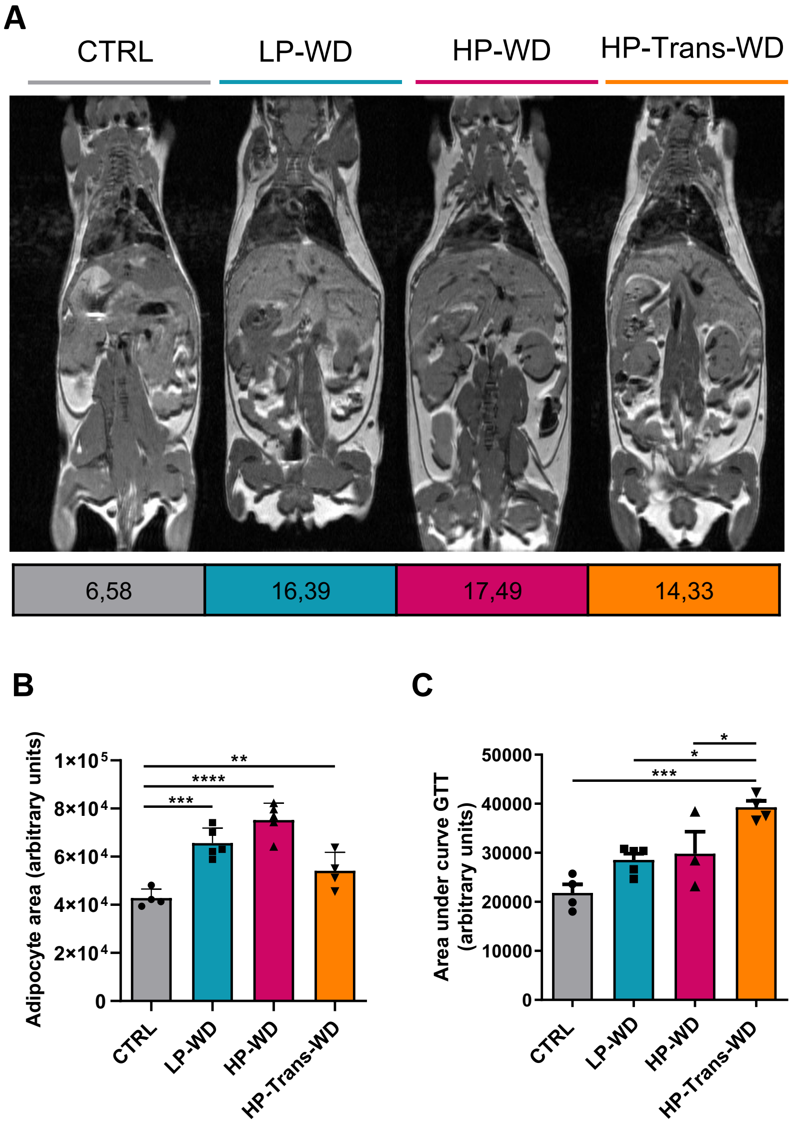
A
CTRL
LP-WD
HP-WD
HP-Trans-WD
6,58
16,39
17,49
14,33
B
Adipocyte area (arbitrary units)
1×10⁵
8×10⁴
6×10⁴
4×10⁴
2×10⁴
0
**
****
***
CTRL
LP-WD
HP-WD
HP-Trans-WD
C
Area under curve GTT (arbitrary units)
50000
40000
30000
20000
10000
0
*
*
***
CTRL
LP-WD
HP-WD
HP-Trans-WD

Suppl. Fig. S1

A

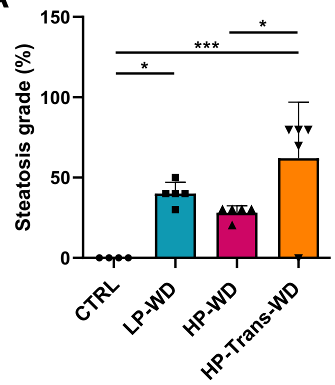

Suppl. Fig. S2

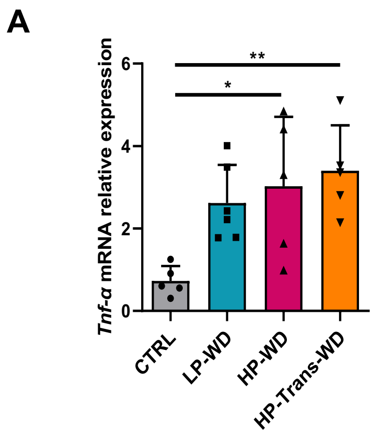
A
Tnf-α mRNA relative expression
0
2
4
6
*
**
CTRL
LP-WD
HP-WD
HP-Trans-WD

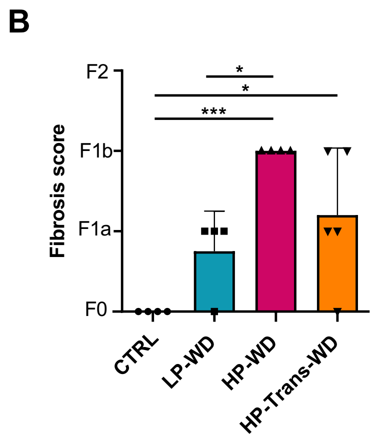
B
Fibrosis score
F0
F1a
F1b
F2
*
*
***
CTRL
LP-WD
HP-WD
HP-Trans-WD

Suppl. Fig. S3

A

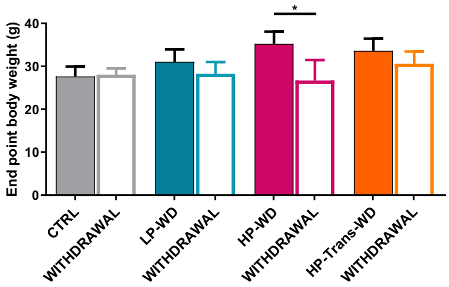

B

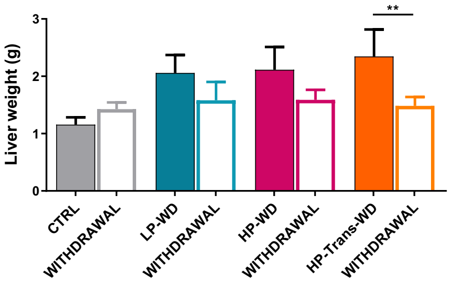

Suppl. Fig. S4

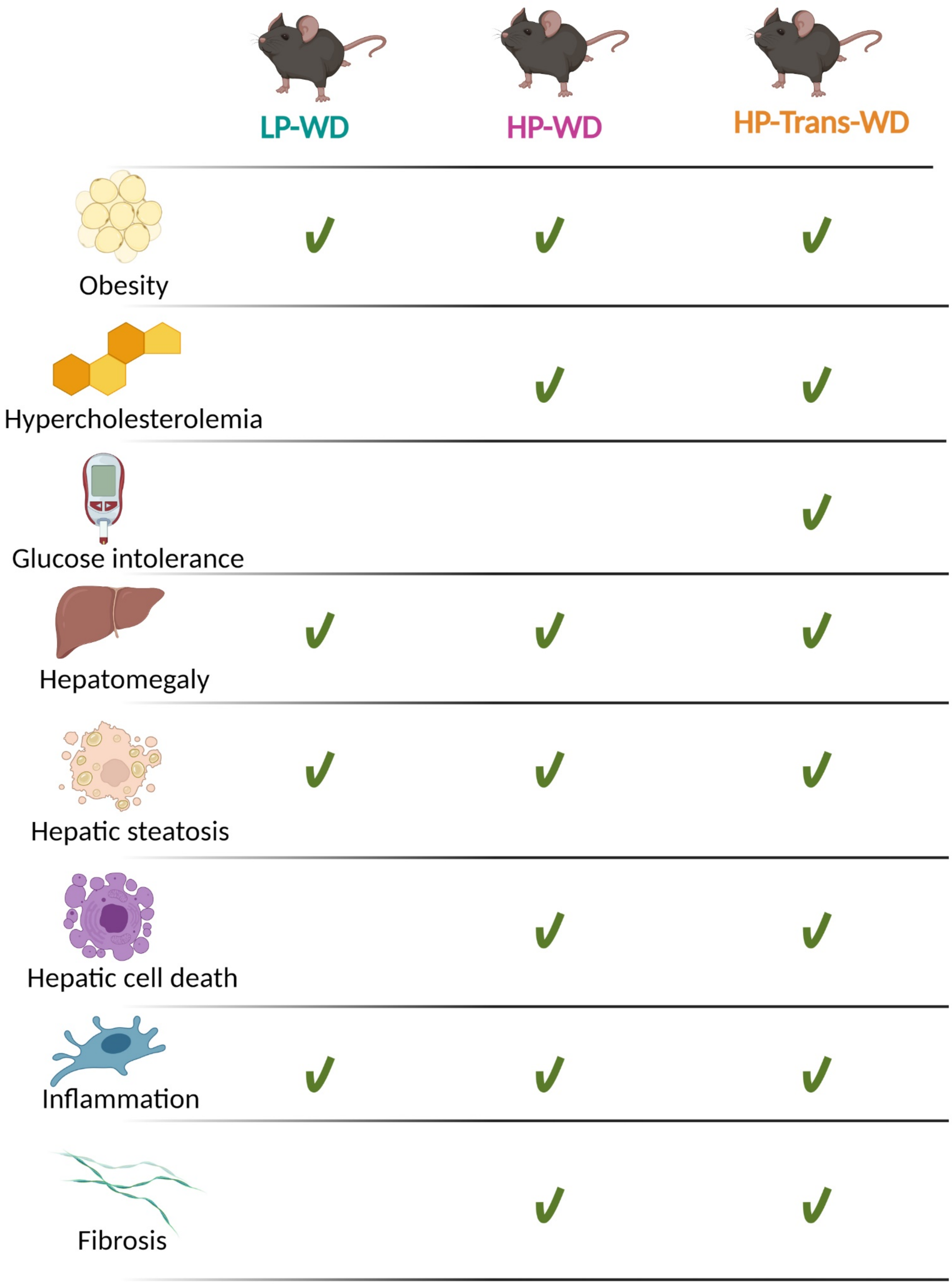
LP-WD
HP-WD
HP-Trans-WD
Obesity
Hypercholesterolemia
Glucose intolerance
Hepatomegaly
Hepatic steatosis
Hepatic cell death
Inflammation
Fibrosis

Suppl. Fig. S5
